# Reminiscences of a European Trip to the International Medical Congress at Moscow, Russia, August 19 to 26, 1897, as a Delegate from the American Medical Association and the Odontological Society of Pennsylvania

**Published:** 1898-10

**Authors:** W. G. A. Bonwill

**Affiliations:** Philadelphia


### REMINISCENCES OF A EUROPEAN TRIP TO THE INTERNATIONAL MEDICAL CONGRESS AT MOSCOW, RUSSIA, AUGUST 19 TO 26, 1897, AS A DELEGATE FROM THE AMERICAN MEDICAL ASSOCIATION AND THE ODONTOLOGICAL SOCIETY OF PENNSYLVANIA.[1]

BY W. G. A. BONWILL, D.D.S., PHILADELPHIA.

It is not at your request I assume this task. Before doing so your attention must be called to an incident in the experience gained when I was a delegate to several societies in Europe, in 1889.

My personal experiences were duly published in the INTERNATIONAL DENTAL JOURNAL. An editor of a western dental publication, in criticising, or rather commenting upon it, took the trouble to count all the "I's," and saw nothing beyond this worthy his attention. This article of the editor was, to me, a lesson which I have never forgotten. Meeting him some time after, he was thanked by me for calling public attention to the *personal pronoun.* No abuse was heaped upon him for this nor for slighting the rest of the article, as there was nothing therein that called for criticism or a passing notice. In all my career, not once has any criticism upon my work been given a reply from me. Such fanning a flame has no good results. Everything done by me has taken quite good care of itself. Time takes charge of such and balances the sheets in the end.

In the present effort to interest you no promise will be made as to how many "I's" will appear. There is some excuse in my case, as the line of work done by me has been unique, and I have had no occasion even to speak of the work of others; hence "I's" will of necessity loom up, and my friend will do me the favor, this time, to simply send me a personal communication as to whether there has been any improvement over the past, and, if he deems it prudent and courteous to an "old stage coacher," to remember that he often places himself in peril when he writes editorials. An old adage, "Oh, that mine enemy would write a book," should ever be kept in mind, even by younger men.

You must not expect me to give in detail the fullest experience of contact with many of my profession, as well as the medical, on a tour of nearly four months. My life was with them almost

---

[1] Read before the Odontological Society of Pennsylvania, March 12, 1898.

solely. It was the intention before leaving home to make a sacrifice of pleasure and live only in an atmosphere suitable to my ambition, and give to my brethren abroad whatever they felt was due from one with peculiar methods of practice, and, by both talk and demonstration, picture to them the instruments as invented, and used in his own way. To this end my dress-suit case was closely packed with dental and surgical engine and every appliance requisite for the programme laid out; which never left my hands from the time I left home, the 30th of July, until my return, 22d of October. Not knowing how the custom-houses in the many countries passed through, and especially on entering Russia, would look upon this enginery and traps, prudence dictated that the case should not go to the care of any other person.

Fortunately, no duty was asked of me and no one suspected me of being a Nihilist.

As to life on the steamer "Werra," there was a medical party of about fifty delegates on their way to Moscow, including several women M.D's., and only one dentist, besides myself, all of whom were strangers to me. The ten days on the ocean were charming, and yet I did not mix with the throng, as so much work had been laid out to prepare for the Congress. Never before had I written on "Pyorrhœa," but the programme of the Dental Section expected me to present a paper. When the essay was done it embraced sixty pages of foolscap. This, with three other articles, on subjects hitherto not made public, consumed much of the time; as also did the revision of one hundred and fifty poetical effusions and the revision of three hundred pages of typewritten matter against Evolution from the assumed stand-point of the Equilateral Triangle. You can imagine when this was done whether I did my share of talking while on the steamer.

Italy, being entirely new to me, caused forgetfulness of dental memories from the hour we landed at Gibraltar and Naples until we reached Milan. In all the principal cities I called upon dentists, but was shown nothing new except a *long white linen gown* worn by a dentist, I think, in Rome, to show his deep and long admiration of the Listerine process of antisepsis. It swept the floor, and one could well suppose that this was his principal garment for this hot climate, and he was surely running the germ theory as nearly into the ground (or dirt) as he could get it. It was an absurd effort to show off antisepsis; the same attempt I found in quite every place visited, several of which will be shown up as the abuse of this over-estimated and overvalued treatment.

In Italy a law has been obtained that will possibly cause every American dentist to leave that country. It compels every one who wishes to practise there to undergo an examination and graduate in one of the medical schools of Italy. There is a bitter prejudice against the majority of American dentists wherever I travelled, with very few exceptions. It was not shown towards me, as I was not looked upon as of that type, but as public property. In every country where I stopped over in any of the cities the receptions given me and attention paid and banquets tendered were seemingly most unselfishly done, and no prince could have had more attention. The effort seemed to be to show to the world that they were ready to receive *any dentist from any country, provided he would freely give of all he knew and demonstrate all his methods of practice.*

We have no reason to object and complain because American dentists are repulsed by the foreigners. The same thing is being done in every State of the United States to prevent our own citizens from practising.

In comparing the capabilities of dentists over the water, and their social standing, with such as we have here, I can say they are on the average far better educated and come from a higher social standard. At least, such as I met.

Very few of the dentists in foreign lands come from the lower walks of life. There is no reason why Americans should be better dentists, physicians, or professional men, except that we have a larger opportunity to develop talent, as the necessity for dental operations is greater than anywhere on earth, and we have a richer people, and the teeth are now better cared for here. Besides, gold is not so much used there as here; plastics have the preference. Prices are not so high, and the patients are more afraid of being hurt. I had a better opportunity, perhaps, of seeing the operations wherever I went than had any other one who ever travelled. I was brought into more immediate contact with the very best elements of professional men, and I can say for them, everywhere, that a more gentlemanly set of men I never met, and never once did I feel any discourtesy, everything being done to make my tour one long to be remembered, with love and attentions from all quarters.

I gave no talks or clinics in Italy. Entered no schools and but few offices, owing to the extreme heat and many dentists being out of town. It was not until I reached Leipsic that I began to realize that I was about to enter upon the course of lectures and clinics promised them through the notices of my intended visit given in the INTERNATIONAL DENTAL JOURNAL and *Items of Interest.* At Leipsic I felt at home. My old student, Dr. Paul Schwarz, and Professor Hesse, of the Dental Department of the University of Leipsic, tendered me every facility to see their school and private practice. As the school was about closing, I had no chance of seeing their methods of teaching and demonstrating, yet I saw enough to satisfy me that to do the right thing by the students they must have better and well-equipped quarters. The school was small, and really their work was more a labor of love, as they could not expect to have large classes with the Berlin school only a three hours' ride from Leipsic.

At Berlin I had been specially invited, before leaving, to meet the Saxony dentists and the Society of Dentists of Central Germany, on the 6th and 7th of August. It was here my first clinic and lecture were given, and for two days my time was fully taken up. Very few clinics were given by the members of the Society. The papers I could not hear, and cannot report upon their value. They were a fine-looking set of men and evidently well educated.

As usual with them, as with us, a banquet came off, and they spared no expense, champagne being the principal beverage for all. They seemed to act harmoniously, and, aside from the banquet after the close of each day's session, a social time was had. They certainly do enjoy themselves and endeavored to have others do so.

Professor Miller was at the meeting, but was unable to take part, and left before the meeting adjourned. He seemed to be in a very precarious condition, evidently from overwork.

I visited several offices in Berlin and the University School, where they have about two hundred students. Professor Miller has not been able to be of much assistance at the school for months. The school was not in session, and of course I saw but little of their methods of teaching.

When in Berlin I called upon Professor Miller, but his physical condition was not such as to warrant a prolonged interview. He, however, asked me to call upon his assistant operator, who would show me his offices and his efforts at carrying out his teachings.

I accepted this invitation, and while I saw some of his patients, yet I found no inducement to change my own simple ways of treatment, which for forty-four years have been satisfactory, notwithstanding all that has been said on the germ theory.

My methods were based upon my father's practice as a medical surgeon, whose teachings and work I had ample chance to observe, until my advent into dentistry in 1854. These were cleanliness, thoroughness, with constant watchfulness to keep down irritation, and, if inflammation followed, to meet each advance and anticipate its ending. With it all he taught me boldness and daring, and to see that all directions were faithfully carried out. The remedies were confined to wood creosote, nitrate of silver, the bistoury, saws, forceps, free incisions, perfect drainage, all deep sacs well opened to inspection; the evacuation of all matter in stomach and bowels, the drainage of the whole intestinal canal by distilled spring water, and the best of food in small quantities, thoroughly masticated without drinks; and, not least of all, water at its various temperatures as an application on all unbroken surfaces; and by all means to see that the hands and finger-nails were clean and well trimmed, and that the instruments to be used be scrutinized, and to have them sharp, as well as free from rust and stain. To this may be added rest and quiet; no meddling with the parts once treated, if doing well; operating with rapidity and as little exposure to the air as possible; binding up wounds in their own blood, first washing with almost scalding water, and to take every precaution against infectious diseases, particularly erysipelatous inflammation, and not to put his own hands on his own person anywhere or allow the patient to touch the parts; the airing of the sick chamber, and permitting no slops to remain. He, in other words, taught me to be conscientious at every step, to not do nor overdo, and nothing more exemplified his treatment than the care he exercised when acting as an *accoucheur.* Now, what more did I see in all my travels to convince me that mortal could do more than follow out these early lessons? And yet I would not decry the work of any who may wish to dive deeply into the germ theory, nor check their investigations. Professor Miller's associate said they were doing no more than these rules I have stated, nor could any one.

One thing I saw wherever I went in Europe, to which I seriously objected. The stationary washstand, with reservoir for cold water alone, and a tub for waste inside, cannot be clean in any sense of the term. Better to have a bowl and pitcher and slop-jar of porcelain on top and in sight. These never can be detected by the finest olfactory senses. While these stands are artistically made, yet if pure asepsis is to be the fad, then those who have earned a reputation through its advocacy should live up to it and thus avoid criticism.

I was thanked for calling attention to this matter. It may not be too late in the day to place this on record as worthy of careful consideration, to enable us to practise somewhat nearer to the ideal direction of bacteriology; and, if we want to be true and pure followers of asepsis, then we should have our floors, walls, ceiling, and tiling of such character that nothing can accumulate. We should wash our dental engine hand-piece every time we shift from a diseased tooth to a healthy one, and, in fact, wash our hands when we remove them from the mouth each time, and never fail to do this should we step aside for a moment to shake hands, handle coin or bank notes, or go into an adjoining room. Everything should be changed,—linen on the head-rest, linen coats, napkins for the mouth,—and a hundred other things must be done to doubly insure against poisoning from any source. Without all this, no one does practise that which he preaches as indispensable. I bow respectfully to all investigations, yet there is such a thing as overstepping the bounds of experience and clinical practice, long since in custom among all clean and conscientious surgeons and dentists.

In justification of this stand taken, there will be found, farther on, the report of a clinic given by me in June, 1897, before the Stomatological Section of the American Medical Association in Philadelphia, to show the value of the clinic over hypothesis, theory, and speculation.

Science is now the dominant fad,—science arrayed against experience and clinical life, and marshalled by a set of men the bulk of whom have no genius for dentistry, and who suppose they can swim with the current started by great thinkers and workers. Such men are not to be followed. I have called attention to the dental school at Moscow as coming nearer the ideal of treatment, as regards rooms and surroundings and the individual wash-bowl of hydrant hot and cold water. I found these at the Guy's Hospital dental school, in front of every chair, compelling the student to be ever on his guard in the presence of the patient. More could not well be done to secure immunity. This once engrafted upon the beginner, all else would follow to secure more perfect surgical work in our profession.[1]

In enlarging upon this let it be understood it is not done in a spirit of criticism and want of regard for the work done in bacteriology, for no one has a higher appreciation of what is being done

---

[1] In a short biographical sketch of Sir Joseph Lister in *Popular Science Monthly*, it will be seen how many changes he made from his initiative treatment and what he now claims to be all that is needed over the all-important, vital axiom,—cleanliness first and thoroughness.

by others outside of my own special work; nor do I wish to have others feel that I do not believe in the application of science to dentistry. No! but from my own practice and all I saw in Europe, whether in hospitals or private practice, or clinics given in both medical and dental hospitals, and the talk on this subject at all the gatherings, I cannot endorse the extravagant directions given for our following.

To show still further that no injustice can come from my talk on antisepsis, I must be permitted to speak of a clinic I gave in Philadelphia, June 8, 1897, before the Dental Section of the American Medical Association. I introduced fifty patients, commencing with the first one for whom I ever practised, showing the result of a mixed action of conservative methods in gold, tin, amalgam, and sectional plates; in fact, as I said, my whole practice was summed up in this clinic, in order to have it criticised in the broadest and to the deepest extent possible by the committee.

Their report was at once given to the main body that the clinic was unique, and without exception the boldest effort ever given in dentistry to exhibit the principle which should actuate every one in freely presenting his work for the criticism of a committee. They did not take exceptions to a single point offered, and thanked me for the privilege given.

If a mechanical invention can be considered to have any aseptic effect in surgery, then I must add to this list that which has already proved the most important adjunct in surgery, next to anæsthetics, if one can believe what Garretson has said of it: "The surgical engine stands next to anæsthetics as the *greatest boon given from dentistry to the world.*" With this instrument alone almost, wounds do heal by the first intention from the perfect freedom from spicula, and saving of time and shock.

In introducing this instrument, I know, from personal experience with both the elder and junior Gross in the Jefferson Medical College Hospital, that the operations I performed in bone surgery, without special aseptic treatment, were perfectly successful; when, if done by hand surgery, Professor Gross admitted to me they could not have been done without erysipelatous inflammation following, and the cure prolonged and probably loss of much bone structure. This engine stands as evidence of how much can be accomplished by machinery irrespective of aseptic treatment.

While I was shown true courtesy from every one, yet it was with difficulty that patients could be seen. This was perhaps not on account of the fears of the operator, but because the patient objected.

In many cases, however, they yielded, when they were told I was an American dentist, and they wanted to show me what could be accomplished in Europe rivalling America; and that, while I was freely giving my time, labor, and love in this mission, it was but fair to reciprocate. I was amply repaid for my vist to Berlin to know that after all the arduous work and research of bacteriologists I was as near to the high-water-mark of success in saving human teeth without practising all the so-called science of bacteriology to assist me. The work performed is futile when it comes to be put into practical use, and especially in the hands of poor operators, as it gives them an excuse for covering up imperfectly performed operations; and, unless an operator is very conscientious, he will become less efficient in mechanical skill and dexterity.

I would not have you understand that I am opposed to the antiseptic treatment and do not carry it out in my practice. I do, but on an entirely different basis. It is well it has been so thoroughly ventilated, for dirt, at one time, ruled the hour in too many dental offices; although, as a class, I think dentists are now more cleanly in their habits.

I have seen so much of asepsis that when applied in cases where immediate action in surgery is demanded, it fails. Tait, a celebrated English surgeon, proved, by his statistics of operation, that he had performed, without any special aseptic treatment in advance, or at the time of the operation, a greater success than Lister with his carbolic spray.

I witnessed an operation upon my own daughter, where two skilful surgeons had diagnosed a tumor of recurrent type, and advised immediate action. The rooms had been cleared of all unnecessary furniture and carpets, walls washed, floors scrubbed, and all the linen and paraphernalia made aseptic, with every precaution for her safety. The case, I was satisfied, and so told the surgeons, as well as my children, was an abscess of strumous character and situated deep down in the axilla, left side, and that the mammary gland was not involved at all. They persisted in first removing the breast, and found no tumor, and then went on the search for it in the axilla, when, to their surprise, an abscess spurted out into the operator's face and all over the wound, spreading the poisonous pus into every tissue, so much so that it was impossible to clean the wound satisfactorily, and it was bandaged up in a doubtful condition; but in spite of all this the wound healed without untoward consequences.

Hundreds of operations are done without any previous treatment and waiting, without any of the means at hand to practise thorough asepsis, and the results are good.

At the American Medical Association meeting at San Francisco, in 1894, an M.D. was exploiting his methods in preparing himself for a case where he was called in hastily to see a woman who was flooding to death. The time consumed was about fifteen minutes before he touched the woman.

A woman M.D. saw a point she could make, and rose to tell her treatment. She said, "I would have rushed into the kitchen, called for soft soap, gone to the hydrant of hot water, and would in three minutes have had my hands perfectly clean and been at my patient's side and saved the risk to her life by the process adopted by the gentleman who has just spoken."

In many places, not only in Europe but in America, have I seen the operators with a woollen coat on in their offices which they had worn for every patient for days and weeks, worn it on the street and wherever they went, while they had tanks of carbolized water as well as bichloride of mercury around the chair to show off their great care for the patient.

*While it is all well to preach and attempt to carry out iron-clad rules, yet there is no treatment that will insure success where the system of the patient is not up to a healthy standard and the operation has not been thoroughly performed, and with scrupulous general cleanliness.*

*With all the application of it to the treatment of pulp canals, the cry is still for further advance. Few succeed.*

As early as 1855 my only medicine in pulp treatment, whether after a live pulp had been removed or in a case of pus formation or an abscess, was wood creosote, and with cotton or zephyr in the canal, after thorough extirpation and rigid cleanliness, and the immediate stopping of the canal and letting it alone. I have no cause to regret that cotton was used or that creosote was my only remedy. No one can show better success and a greater number of human teeth preserved without pulp exposure, or after exposure, from this simple treatment.

Antiseptic treatment has done a wonderful work, and all praise to the agitators! But let us not lose sight of the fact that with it all our operations in the various branches of our art would not be worth a farthing without mechanical engineering details are carried out faithfully, and certain physical obstructions are removed and *all stagnation is displaced.*

In Vienna and Venice our stay was so short that, further than sightseeing, no dental experience was had. It was not until Moscow was reached, the objective point of our trip, that life began to take on the phase of hard work.

I cannot pretend to describe to you the old city of Moscow, with its long and interesting history, nor can I give you what I saw at the International Medical Congress, as my time was consumed in the work laid out in the clinics, which very wisely was planned to get the most good from the meeting, as the reading of the papers alone would not have taught much without clinics. To begin with, it was the dirtiest city I think I ever visited, and yet the dwellings and public edifices inside are marvels of cleanliness.

The Dental School in Moscow was managed by private dentists, and it was here our clinics were held, and I can say for it that I never saw handsomer rooms and a school where asepsis came nearer to being accomplished, so far as the buildings and equipments were concerned, combined with clean operators.

The faculty were fine-looking men from the front ranks of society. But few, if any, enter the dental schools from the lower grades. There are but two grades, the high and the low, and, of course, the men who enter any of the learned professions come from the higher. There is no middle class. Hence the men who are found in dentistry in Russia must be better educated and adapted for their work.

There was one dental school in Moscow. In Warsaw there was a dental college and with much larger classes, numbering two hundred students. That in Moscow had but eighty students. The sessions had closed for the summer. In Warsaw I did not get a chance to even peep into their college building, although I afterwards met the Dean and his son at the Congress, whom I found men of character and evident ability, and who spoke the English language fluently, as did many Russian dentists at the Congress.

Four hours each day for four days were given up to the clinics, and, as I said before, it was a comfort and a pleasure to operate in the palatial rooms of this dental school.

As to what was done by others I could not tell you, as not a moment was given me to look around at the work of others. My countrymen, Drs. Younger and Talbot, read papers; and I think Dr. Younger showed his practice in pyorrhœa and perhaps a case of implantation. Talbot talked about etiology and degeneration and visiting hospitals.

There were many devices shown and methods of practice from dentists in Europe, but withal I cannot recall anything new of any value. My special attention was called to this later on, after clinics.

The clinics were crowded every day, and attention was held without abatement, giving apparently more satisfaction to see work done than to hear it talked about.

My own clinics were in the mouths of living patients. Four hours a day for four days and constant talk all day.

Abbey's cohesive and old-fashioned gold-foil were used with electric and mechanical mallets. Abbey's soft foil was shown to be capable of being impacted solidly and perfectly with smooth points only, yet it was easily done by hand-pressure. It was shown to have softer qualities than any other foil, and would save tooth structure without recurrence of decay.

My second clinic was with amalgam, showing the new matrix made of a rubber dam clamp and modelling wax or gutta-percha, and how all of the mercury can be absorbed from the hardest filling made under the pressure of bibulous paper by rubbing into the surface of each filling, before the matrix is removed, the chips of amalgam until no more can be rubbed in. Also, how gutta-percha can be used for matrix for gold filling.

I gave an oxyphosphate clinic, and explained how paraffin saves it from destruction. A clinic also on the many and vast uses of gutta-percha, and also a clinic on new method of bridging, with clasps and made removable, as well as bridging permanently without gold caps. I gave them a synopsis of clinics and methods (to be published elsewhere, and in the proceedings of the Congress).

It would take too long to enumerate all that was done to fill the hours completely each day. They had become enthused over gold caps and bridges, cataphoresis being hailed with delight, and while it had not been largely used, yet it was soon to be tried, and I gave them my paper on it.

I had an opportunity of seeing into the mouths of many persons at the clinics, and invariably decay had exhibited its ravages as thoroughly as I had witnessed it in cases in my own practice. Not only in Russia, but in every country, degeneracy looms up wherever civilization has stepped its foot, and there is no greater mark of failure of life in its physical aspect than the prevalence of caries among all classes. Luxury has had its effect upon every nation.

As I before stated, so constantly was my time engaged that I had little chance of seeing the clinics of others. Out of seven thousand delegates not more than two hundred were at the meetings of the Dental Section; and, judging from the badges, not many were delegates from organized societies. From the pen of a Russian writing upon the status of dentistry in that country to the *British Journal of Dental Science*, I would read you some extracts if time permitted.

The general Congress was no doubt a success in all its departments, yet no marked treatment or advance was brought forth. There was a surfeit of papers and many that were only read by title. The Surgical Section particularly was the most active, and it was into this that our Dental Section was merged, which gave it more character, although our work was entirely distinct from all others.

I cannot close my remarks about the Congress without a word to show you how well our Dental Section was treated. Aside from having our proceedings to ourselves, as all the other sections had, when it came to the grand banquet on Friday evening the Dental Section was as highly honored as any other section, having its table in the midst of the medical men from all over the world. At the table just back of me sat the ex-President of the American Medical Association, Professor Senn, from Chicago, with several medical gentlemen from America and elsewhere, and he can tell you whether the men at our table contrasted favorably with the others and how they treated your essayist to show they were not unmindful of the advances made in dentistry as fully equal to any made by the medical profession, and that they were proud to do justice to America and make this banqueting season an occasion when they could demonstrate their interest in the representative from your country. I felt it a great privilege to have been present on that occasion.

That you may know how the Americans were treated at the International Congress, I have here a letter to me from Professor Nicholas Senn of Chicago, the President of the last American Medical Association which met in Philadelphia in June last, who went over on the same steamer to attend the Congress. Inasmuch as I was a delegate from this society, as well as from the American Medical Association, I can be pardoned for showing Professor Senn's letter.

Aside from the honors spoken of here, many diplomas of honor have been sent me since my return.

The French School of Dentistry and the French Dental Society gave me a very large and handsome silver medal, while the Dutch Dental Society also gave me, at the close of my clinic at Amsterdam, a portfolio of thirty photogravures of this quaint old country, measuring two or three feet.

When I stopped and gave clinics and talks, some memento was given to remind one of the love that exists everywhere for a fellow-dentist who will unselfishly go to a clinic and teach all he may know.

While I was not in Spain, yet it was my good fortune to have the almost constant society and attention of my friend, Dr. Aguilar, of Cadiz. Speaking, as he does, several languages, made my work far more effective; and having been a great traveller himself, he, in reality, was my guide from Berlin to Moscow, and on my return to Hamburg. He is fully alive to the wants of our profession, and is to be found wherever any great meeting is in progress. He was sent as a special delegate from the Spanish government.

It would be impossible to speak personally of the men who left nothing undone wheresoever my pilgrimage took me. To feel a lasting debt of brotherly love ever welling up within me is all that can be returned to repay the debt until they come to America, or I can contrive something to aid them in their work.

This manly letter of Professor Senn will sink their stupid selfishness to honor their own men:

"532 DEARBORN AVENUE,
"CHICAGO, February 25, 1897.

"MY DEAR DOCTOR BONWILL,—Permit me at this late date to congratulate you on the recognition you received in all parts of Europe on your trip to the International Congress last year. It is a source of pride to me that so many honors were conferred upon a well-deserving American delegate. I thought I worked hard, but I have found more than a counterpart in you.

"Hoping that your life and health may be spared for a long time for the benefit of science and humanity, I am most sincerely your friend and admirer,

"NICHOLAS SENN."

"532 DEARBORN AVENUE,
"CHICAGO, March 13, 1898.

"DEAR DOCTOR BONWILL,—Many thanks for your very appreciative letter. I am sure in saying what I did I only voiced the sentiments of the whole Medical Profession. Next time you are in Chicago, I must see more of you. It will please you to know that the patient upon whom you saw me perform a very difficult operation for cancer had no unpleasant sequela.

"I do hope I will meet you in Denver.

"Very sincerely yours,
"NICHOLAS SENN."

It was a mark of the nobility of Professor Senn to voluntarily pay me such a compliment as a medical man to a dental delegate, when it could have been a cause for deep jealousy against our Dental Section at the Congress or at the American Medical Association meeting. I am sure dentists at home can well feel complimented at the letters of recognition coming, as they do, from so high an authority.

This is also in marked contrast with the action of the four honorary presidents at the Berlin Congress, where I was specially invited to read an article on Articulation. These men informed me I must not go to the Congress in 1891, or they would make it very unpleasant for me if I did go. I remained at home and waited my opportunity, which came at the Congress at Moscow, and, instead of dishonoring my profession, I am sure they cannot complain at the honors given through me to them. If the dental profession here want to gain the highest recognition by the medical fraternity in their Congress, they must learn to respect and honor and uphold their own men, and not exhibit petty jealousy towards those whom the world acknowledges have done something for progress. I can afford to pardon such work, as I have always known opposition from the first; yet, as all things come to him who waits and labors, so I have tried to do, and I am none the weaker from that course. I hope yet to do what will always help my calling to rise above itself and its own follies. My love for it buoys me above all opposition to drive on.

The following letter from a Moscow delegate will speak for itself:

"Office of 'Pacific Medical Journal,'
"San Francisco, Cal., April 15, 1898.

"Dr. W. G. A. Bonwill, 2009 Chestnut Street, Philadelphia, Pa.:

"Dear Doctor Bonwill,—Ever since the receipt of your kind letter of the 11th of January, I have hoped that within a few days I should be able to write to you about my talk before the Dental Society. For one reason or another something has intervened to prevent my appearing before this Society. The time has at last come, however, when I can tell you definitely what I am expecting to do. I have received an official invitation from the California State Dental Association to give an address at their next annual meeting in June. I have accepted the invitation and propose to make you and your work the chief subjects of my paper.

"One week from next Tuesday I shall, also by invitation, give a short preliminary talk to the local Dental Society. Last month at a banquet given to Dr. Brophy of Chicago, Dr. Barrett of Buffalo, and Dr. Hoff of Ann Arbor, I was invited to say a few words. I improved the opportunity, in speaking of American Dentistry, to call up your name. I told these gentlemen that in the reception accorded you in Moscow and other cities of Europe, American dentists were highly honored, that through you a high tribute was paid American dentistry.

"I take every opportunity to bring your name forward, as I feel that it was through my acquaintance with you and hearing you talk about dentistry that I for the first time realized the tremendous advance made in the science of dentistry during the past twenty or thirty years. I became convinced that the science of dentistry is as mu :h a part of medicine as is ophthalmology, otology, or the special study of any other part or parts of the human economy. They are co-equal and cannot be considered solely as organs having no connection with the rest of the organism. I take pleasure in dwelling upon this idea that the doctor in medicine and the doctor in dentistry are making towards the same end,—viz., the conservation of health. It is not necessary for any of us to make a point on the superiority of one branch of medicine over another. To my mind it does not exist. Our only reason for devoting our lives to a single branch of medicine is due to the limitation of human knowledge and our inability to master the whole range of medicine. I propose in my address to develop somewhat a few of these ideas.

"Very sincerely yours,
"W. F. Southard."

At St. Petersburg I was met by the dentist to the Czar, Dr. Wollaston, an American from Pittsfield, Mass., who insisted on my remaining several days, when he would show me the wonders of that modern city and perhaps get me a glimpse of the Czar; but I had to forego this pleasure and hasten to Copenhagen. The time was so limited after the Congress that while I was telegraphed to go to Christiana and Gottenburg to meet the dentists of Norway and Sweden, with the proffer of a good time, yet I had to rush on to Stockholm, where so many of the dentists spoke English fluently and made my visit far more agreeable, although I did not want for interpreters wherever I went. Dr. Aguilar, of Spain, was almost constantly with me. I had the advantage, however, of being able to clinic, and that would have conveyed a language unmistakably plain.

The dentists of Stockholm were markedly attentive, and threw everything aside to make my stay pleasant and profitable. They were a wide-awake set of men, and their social position was such as to make their profession worthy of their following. They were well educated and with a live dental society and dental rooms, where, as a club, they could enjoy life after their labors. Their museum and library were full; I saw many handsome offices, and their homes showed a refinement that dentists should everywhere strive to obtain.

Their annual fair was under way, and I was escorted there several times, and twice a dinner was given me on the grounds. This fair gave the best of evidence of the ingenuity of the Swedes. It was made up almost exclusively of the productions of the natives, and you can imagine that with the advantage of such a school every year, and the high character of the Swedish people, much must be expected from the native dentists, and that they could well do without American operators to help them. From what I saw there, I could well feel that here were men worthy of our admiration, and such talent should not be intruded upon, as they are well able to look out for themselves and their countrymen in our professional work.

When one goes into such life, he feels the good honest influence that is being constantly exerted by the people everywhere and that dentistry has good cause to feel honored by men who were true to their calling, true to each other, and true to one from across the sea.

At Copenhagen was held a repetition of clinics, talks, lectures, dinners, and social attentions.

The native dentists are a band of brothers, although in every city I found contending parties and cliques. It was enough for me, however, to see their offices, instruments, surroundings, and to hear them talk, to judge of their capability. No new method of practice was revealed, yet they were fully abreast in the treatment as given by us and others in the text-books. Here, as everywhere I went, the cry was for some plastic for filling, and for some method to prevent recurrence of caries, and a plan that could be followed that would insure what had been done by the operators against the ravages of time. No form of gold had been found that would fill the demand, nor had any plastic, and they were ever ready to hail with delight any one who would offer anything equal to the demands of the hour, and for those who desired to honorably practise dentistry.

Here, as in quite all of Europe, they are handicapped by low prices, and when I tell of my visit to Dr. Herbst at Bremen, I will show, from his experience in the manufacture of submarine gold-foil, the true condition of affairs all over Europe.

The Copenhagen dentists show that they practise a profession with love and a desire to leave nothing unknown that will aid them; and, above all else, they are so acting that the social status shall rank as high as that of the surgeons. I shall ever feel proud to shake such men by the hand when they come to America.

You must know that it is no trifling effort for a man of sixty-four to lug all his traps around in a dress-suit case heavy enough for a galley slave, and unpack and pack up after each clinic, and talk incessantly for three months. But clinics pleased them everywhere more than anything else, and the effort is always appreciated and draws crowds that no lecture will. It was a labor not for myself, but to honor my Society and the American Medical Society; and whether you say, "Well done, faithful servant," or not, I have performed a duty for which home recognition offers least remuneration.

After such recognition I was loath to leave, although I needed rest, which was now to come for a few hours in the enchanting steamer that was to carry our Cook medical party to Hamburg, where at last I had to bid adieu to them after the long tour from Gibraltar all through Europe. But many of them I have met since, and memory will ever cherish associations thus made, and which are the more refreshing and lasting as time passes.

At Hamburg a delegation met me at the cars who had long ago extended a special invitation while I was at Berlin. The same routine of talk and clinic was repeated. Social attention here was a marked feature, and the bowl was always brimming full, and warm feelings overflowing at the home of every one where I was invited. The live questions were brought up for discussion, and deference was paid to your delegate and attention given his efforts to instruct.

It was here that a gentleman of wealth gave fifty thousand dollars to the city to establish a dental infirmary for the benefit of the worthy poor, and eight thousand dollars extra yearly to keep up the expenses and to pay several operators a salary that good services could be had. This was an institution worthy the pride of any city.

It was at the suggestion of one of the Stockholm dentists that it was founded. The dentist asked the wealthy donor for ten thousand dollars, and he told him that was not enough, and at once gave him fifty thousand dollars to have a building put up that would meet every requirement. He had made it a model in every way and a most worthy benefaction.

I met with no set of dentists who gave so much evidence of brotherhood and hand-to-hand work for the elevation and promotion of their art.

Let me say here, that there was no one thing upon which I held forth to instruct that gave them apparently more pleasure in the knowledge acquired than the laws of articulation. I was made to show up this part of my teaching more than all else at every point where I stopped over, and, be it said to their credit, they absorbed it more perfectly than the American dentists at home had done in thirty-six years, and for a very good reason: that more of them are academically taught in polytechnic schools than the bulk of the profession in America. But what I wanted so much to see was deprived me. Few of the operations performed by them were shown me. They could tell their desires and wherein they had failed, and their longings for higher and more general knowledge.

What I have told you of all other places answers well for Hamburg. With a view of their magnificent harbor and their appreciation extended I passed over to Bremen to see my old friend, Dr. Herbst. He had sent me a special invitation before I left America to be his guest on my return from Moscow. He surely gave me a warm reception and one that was in response to the manner in which we all treated him while in America several years ago, when it will be recalled he was brought by his personal friend, Dr. Bödecker, to show me the rotation method of filling the teeth with gold. He had much to show me, but what was now uppermost in his love was submarine gold.

While he still uses rotation, yet he has not the necessity for it as formerly. He showed me a great many cases in his practice previously done with submarine gold, as well as how he manipulated it and where he used it. It is a gold he is manufacturing, and of which, he informed me, he had sold much in Europe, but none in America, and wanted me to interest myself in his behalf in this country.

For what purpose has he prepared this? What are its virtues and peculiarities? What his method of insertion? et cetera. I asked him what excuse he could offer for such treatment. Did he consider it good practice to teach men submarine work instead of instructing them to keep the gold dry? It was sufficient for me to see him manipulate it by hand, and then with the burnisher in engine. First, he made no pretension to giving the cavities the proper shape to prevent recurrence of caries; used a matrix of peculiar design of his own, which he hastily placed on, never using the rubber dam; taking no precautions to keep the cavity from becoming submerged or to have the gold remain dry; in fact, he exulted in calling it submarine.

All compound cavities he converts into one of four walls. The gold is used in the form of cylinders and carried from the bottom, overlapping the walls and wedging in finally from the centre, and then compressing as in the old style of soft gold and hand-pressure of fifty years ago. Many of the operations I saw him perform he could have easily kept dry; but no, it must be submarine.

I told him there was nothing new about introducing gold under saliva or water. There were several fillings in my own mouth, put in by me forty-two years ago with Abbey's soft foil, that I could not keep dry for a moment, and which are still doing service. I

told him of a clinic I saw Professor Peirce perform before his class, injecting water in the cavity all the time. But while it can be done, and substantially so in many cases, yet it will never do to teach men this thing, as it would soon render them incapacitated for any fine operations, and make of them sluggish and very indifferent operators. I was frank to say to him that he was doing a wrong thing to his patients and the profession and himself. On every approximal wall and space the food would crowd in, and there was sure to be a recurrence of caries. In no instance could he make a contour, and never any wide interproximal spaces such as I make in plus contouring, and it would be impossible to call such operations saving ones. I asked him what excuse he could give. "Well," he said, "in Germany we cannot get more than from two dollars to four dollars at most for gold fillings, and for amalgam one dollar to two dollars," and as he could not afford to practise for such prices, he was compelled to put in as many gold fillings as he could with this submarine gold in order to meet the expenses of his establishment. Besides, he claimed that it should be adopted by every one, and it was being used by some of the best European dentists, since it overcame so much preparation for cohesive foil and did away with the rubber dam; and also gave his rotation method another chance for extended life; and, withal, helped to do away with amalgam, gave assistance in making more revenue and labor not so great, and more patients were benefited for a short time and without pain. He certainly felt all he said, and gave me his gold to try.

All these excuses or palliations in practice could not turn me away from what forty-three years had told me was wrong *in toto*. Yet there are cases that come to me where gold would seem indicated from position only, and where once I placed it in wet; but I will no longer do so, as we have other materials now at our disposal. It can surely be reached in the use of amalgam in all teeth posterior to the cuspid, whether wet or dry, and the success attained will show that it is impossible to tell whether it was placed in wet or dry.

I begged him (Herbst) to relinquish his idea, for such a practice would not be recognized in America among the best of men, and his reputation would be smirched and his prospects in the near future injured, for it would not pay in standing or financial results. He was dead set against amalgam, and seldom used it, not for want of valuation, but, as I said, because there was so little profit in it.

His courtesy and that of his family I cannot forget, and his efforts in having me for four or five days at his office was generous and kind, considering how few dentists would allow any one to enter before a patient. I was pleased to learn in many ways that his ingenuity as a dentist was appreciated by the city, although he has his strong opponents and his rotation method is not generally used in Europe. He is a hard worker and a worthy man, and he has the talent to practise our profession if only centred and balanced in the right methods to be adopted. He could do it, as he is not only a mechanician, but good at improvising, enabling him to overcome difficulties that confront every one of us.

Dentistry can never rise in any country where prices are, as here, kept at the "low-water-mark." A dentist "is a man for a' that," and must feel he is paid for his brains, or he will finally become a mere tool.

I saw very little of the dentists of Bremen, as my visit was consumed quite wholly with Dr. Herbst. I did visit several there, but, aside from seeing their offices, gained nothing from their patients.

I regret that I have felt obliged thus to criticise, for I estimate the man. With a hearty shake of his hand, after having our photograph taken together, at his request, by a woman artist (a good one she was), which I present you here, my mission was now to proceed to Amsterdam, which was somewhat out of direct line to Paris.

It was in quaint old Amsterdam where the wind-mill rears its head on the broad plains where the winds have such a fair sweep. Dr. John E. Grever, the President of the Dutch Dental Society, had written me in advance of my arrival at Milan, Italy, and at Berlin the Society was represented. He was also delegated to give me a pressing invitation on my return from Moscow, and while there the day was appointed when I would surely be present. It was upon a Sunday, and dentists from all parts of Holland came for the special occasion. From eleven A.M. until five P.M. it was one continuous talk and demonstration, and their attention was certainly unique and most gratifying and stimulating to your delegate, who was beginning to feel it was time to hold up. Dr. Grever speaks English fluently, as do also all the Dutch dentists, and his assistance and attention was so courteous and full that my labors were far less than they otherwise would have been. It was at his house I was entertained for over a week, which gave me a chance of seeing more of his patients and his work than almost anywhere I had been, and it was gratifying to me, because all that

I saw of him and his practice evidenced conscientiousness in what he was doing.

His zeal was such that he was anxious to have the Dutch boys and girls see an American, since he had several times met me at home and in Europe, and as their President he had certainly exercised himself to get up this meeting.

I cannot pass on without mentioning that six or more young lady dentists were present, and, to their credit be it said, they were most favorably looked upon by the men of Holland. They were surely a handsome set of women, and showed the intelligence in their faces that marked their qualities of character and ability.

One in particular was the handsomest woman I had met abroad, and on visiting her afterwards at her home in the Hague I found she was highly educated, spoke several languages, was a fine mechanic, well posted, so far as I could quiz her, in every branch of our art, and practised with an enthusiasm no man could excel, and anxious still to learn how others practised. She was educated by her father, whose social position (and as a teacher of languages and belles-letters in a college near her in Holland) showed it had not been lost upon her. The day and evening I was in her offices I saw many of her patients, and can speak well for her future. One thing made her practice unique,—she would not practise for men, only for women and girl children. I told her she had good sense, and she would be most likely to keep a high reputation and have plenty to do. I gave her an invitation to visit America, and I would give her three months, if she wished, by my chair, feeling that such a woman dentist was well worthy my attention and commendation, for she was certainly well equipped in every way, and, withal, in four years had a practice of four thousand dollars, running her own establishment and educating her brother in dentistry, who would be sent to America for his finishing touches and finally to be her associate. I did not get a chance to visit the offices of many dentists at Amsterdam, on account of sight-seeing and, in addition, a clinic before the surgeons and staff of the university there with the surgical engine.

As to the beauties of this old city, I cannot speak here, but simply say no city in Europe pleased me more, and I left without seeing much of the life, the scenery, or its institutions. The time was quite all devoted to the dentists. To do justice to this one place, and my impression of the dentists of Holland, would consume many pages of paper.

The banquet given me after this Sunday meeting was certainly gratifying and gorgeous, and a fitting conclusion to that long day of labor and love. My friend, Dr. Grever, thought he would please me beyond degree by having one of my articulations carved from a huge turnip brought in on the dish of ice cream dessert. It was very nice in him and not to be supposed that the bloodless turnip was to represent the true value of my pet invention. While I rather hesitated to go out of my way to visit Holland, after so long a tour and needing absolute rest, yet it was a red-letter week, and if I only did enough for them to pay for the attention received from them I shall be content that I did not pass them by.

With reluctance, I now bade adieu, to speed on to Paris.

(To be continued.)

